# Safety and Efficacy of Split-Dose Thiopurine vs Low-Dose Thiopurine-Allopurinol Cotherapy in Pediatric Inflammatory Bowel Disease

**DOI:** 10.14309/ctg.0000000000000544

**Published:** 2022-12-08

**Authors:** Lucia Cococcioni, Licia Pensabene, Maria Giovanna Puoti, Sara El-Kouly, Sibongile Chadokufa, Raechel Buckingham, Edward Gaynor, Efstratios Saliakellis, Fevronia Kiparissi, Osvaldo Borrelli

**Affiliations:** 1 Gastroenterology Department, Great Ormond Street Hospital, London, UK;; 2 Paediatric Department, “V. Buzzi” Children's Hospital, University of Milan, Milan, Italy;; 3 Department of Surgical and Medical Sciences, Magna Graecia University, Catanzaro, Italy.

**Keywords:** pediatric inflammatory bowel disease, thiopurines, split-dose thiopurine, low-dose azathioprine-allopurinol cotherapy

## Abstract

Split-dose thiopurine and allopurinol-thiopurine cotherapy strategies have been suggested as rescue therapeutic options for children with inflammatory bowel disease (IBD) and impaired thiopurine metabolism. We compared the efficacy and safety of these regimens in patients who previously failed conventional thiopurine treatment.

**METHODS:**

Children with IBD treated with split-dose thiopurine or low-dose thiopurine-allopurinol cotherapy were retrospectively identified. Medical records were reviewed for demographics, treatment regimen, reason for thiopurine failure, side effects, and discontinuation of treatment. Laboratory findings were evaluated at different time points.

**RESULTS:**

After prior therapeutic failure, 42 patients were on split-dose regimen (group A) and 20 patients were on thiopurine-allopurinol cotherapy (group B). Twelve patients crossed from group A to group B because of treatment failure, 1 patient was lost at follow-up, and 1 patient discontinued the treatment. The final cotherapy group comprised 29 children (group C), while the split-dose group (group D) included 31 children. Intention-to-treat analysis showed significant differences between split-dose regimen and thiopurine-allopurinol cotherapy for 6-thioguanine nucleotide (6-TGN)/6-methyl mercaptopurine (6-MeMP) ratio (*P* < 0.001), 6-TGN (*P* < 0.05), and 6-MeMP (*P* < 0.001) at 1–3 months. As per protocol analysis, there was a significant difference between group C and group D at 6 months for 6-MeMP (*P* < 0.05) and 6-TGN/6-MeMP ratio (*P* < 0.05) and at 12 months for 6-MeMP (*P* < 0.05) and 6-TGN/6-MeMP ratio (*P* < 0.001). Side effects were more frequent in allopurinol-thiopurine cotherapy (*P* < 0.05).

**DISCUSSION:**

In children with IBD and impaired thiopurine metabolism, split-dose thiopurine and low-dose thiopurine-allopurinol cotherapy are both effective therapeutic strategies. The latter shows higher efficacy but a higher side effect rate, suggesting the use of split-dose regimen as the first-line approach.

## INTRODUCTION

Thiopurines, both azathioprine (AZA) and 6-mercaptopurine (6-MP), are commonly used to maintain remission in children with inflammatory bowel disease (IBD). However, up to 40%–60% of patients eventually discontinue this therapy because of lack of efficacy or adverse events (1).

Thiopurines are normally converted through numerous enzymatic steps into the pharmacologically active red blood cell (RBC) 6-thioguanine nucleotide (6-TGN), which represents the predominant active metabolite responsible for thiopurine therapeutic efficacy and correlates with the maintenance of remission in patients with IBD (2). However, elevated concentrations of RBC 6-TGN are associated with bone marrow suppression (2). On the contrary, thiopurines can be inactivated by thiopurine methyltransferase (TPMT) into 6-methyl mercaptopurine (6-MeMP), which is associated with increased risks of side effects, such as hepatotoxicity and myelotoxicity (3–5).

TPMT genotype and enzymatic activity have been shown to influence the 6-TGN and 6-MeMP levels and a significant portion of individuals with the “normal genotype” exhibit preferential 6-MeMP metabolism (6). These patients, called “hypermetabolizers,” may metabolize thiopurines in a skewed fashion, leading to high RBC 6-MeMP concentrations, often paralleling “subtherapeutic” level of 6-TGN (6). These patients are usually identified by a 6-MeMP/6-TGN ratio>11, which is associated with a lack of therapeutic efficacy and an increased risk of developing hepatotoxicity (7). In this subset of patients, escalation of 6-MP or AZA dose to reach therapeutic range of 6-TGN often results in side effects, including dose-dependent leukopenia, transaminitis, and/or flu-like symptoms (headaches, nausea, myalgia, fatigue, and general malaise) (6).

Among the strategies to optimize thiopurine therapy (administration of thioguanine or coadministration of 5-aminosalicylates), either a low-dose AZA-allopurinol cotherapy (LDTA), consisting in the reduction of thiopurine to 25% of initial dose with addition of allopurinol, or a split-dose of thiopurine (SDT), consisting in splitting thiopurine dose from once to twice a day while keeping the total daily dose the same, has been also suggested. However, whether these strategies are effective and safe for normalizing thiopurine metabolism and which one is the most effective and safe in pediatric population is still a matter of debate. In this study, we aimed to investigate the effectiveness and safety of LDTA compared with that of SDT regimen in a pediatric cohort of patients with IBD (pIBD).

## PATIENTS AND METHODS

### Study design

We conducted a retrospective study trough electronic medical records of all children with IBD followed up at the Gastroenterology Department of Great Ormond Street Hospital of London from 2010 to 2021. Inclusion criteria were as follows: (i) younger than 18 years during intervention; (ii) diagnosis of IBD; (iii) treatment with thiopurine; and (iv) a record of pharmacological intervention to normalize thiopurine metabolites. All patients who switched from conventional thiopurine treatment (weight-based, once-a-day fashion) to LDTA or SDT because of preferential 6-MeMP metabolism or side effects were enrolled in the study. Thiopurine drug monitoring has been routinely performed in all patients with IBD in our institution. Our data sources were the pharmacy and administrative patient databases at our institution and the IBD databases maintained within the department.

Diagnosis of Crohn's disease (CD), ulcerative colitis (UC), or IBD unclassified (IBD-U) was determined by IBD specialists using standard clinical, radiologic, histological, and endoscopic criteria according to international guidelines (8,9). AZA and 6-MP doses were adjusted according to TPMT activity and 6-TGN levels. The initial dose of standard therapy was 2 mg/kg/d once a day increased up to 3 mg/kg/d once a day for AZA and 1 mg/kg/d once a day increased up to 1.5 mg/kg/d (max 75 mg) once a day for 6-MP, accordingly to thiopurine metabolite levels. If TPMT genotype was heterozygous or TPMT enzymatic activity was moderately low, AZA was reduced to 1 mg/kg/d once a day and 6-MP was reduced to 0.75 mg/kg/d once a day. Preferential 6-MeMP metabolizers were defined as patients on maintenance therapy with AZA or 6-MP whose RBC 6-MeMP levels were > 5,700 pmol/8 × 10^8^ or 6-TGN < 230 pmol/8 × 10^8^ or 6-TGN/6-MeMP ratios > 11 (7).

If thiopurine metabolite levels did not normalize despite adjustment accordingly to TPMT, the conventional treatment was switched to either LDTA or SDT regimen. If the first-line pharmacological intervention failed, the treatment was switched to the other regimen.

Demographics, disease phenotype (CD, UC, or IBD-U), TPMT genotype and/or phenotype status, and reasons for LDTA or SDT intervention were collected at baseline. Baseline was defined as the starting time of LDTA or SDT regimen. Blood tests (alanine aminotransferase [ALT], full blood count, C-reactive protein, erythrocyte sedimentation rate, and albumin), fecal calprotectin, drug metabolite levels (6-MeMP, 6-TGN, and 6-TGN/6-MeMP ratio), concomitant medications, clinical disease activity, and side effects were collected at baseline and after 1–3, 6, and 12 months. Disease activity was assessed by pediatric UC activity index or pediatric CD activity index for UC and CD, respectively, and by physician global assessment (PGA) for all patients with IBD. PGA was scored as remission, mild, moderate, or severe disease.

### Treatment regimens

Patients were divided into 2 different groups according to the first undertaken pharmacological intervention: group A (SDT) and group B (LDTA). In group A, the dose of thiopurine was split into 2 doses per day, while in group B, the dose of thiopurine was reduced to 25% of the initial dose and combined with 50 or 100 mg of allopurinol once a day according to patient weight less or greater than 30 kg, respectively (10).

### Evaluation of Patients and Study End Points

Treatment efficacy was defined as normalization of thiopurine metabolite profile at 6 months from treatment intervention according to the following parameters: 6-TGN: 230–450 pmol/8 × 10^8^ and 6-MeMP <5,700 pmol/8 × 10^8^ with 6-MeMP/6-TGN ratio <11 (11,12). We considered also effective the treatment leading to the resolution of thiopurine adverse effects.

Treatment failure was defined as the failure to reach the abovementioned adequate levels of thiopurine metabolites after 6 months from pharmacological intervention or persistence of side effects. Side effects related to thiopurine therapy included the following: hepatotoxicity, defined as an increase in transaminase levels more than twice the normal upper limit; myelotoxicity, defined as leukopenia (white blood cells <3.5 × 10^9^/L), neutropenia (neutrophils <1.5 × 10^9^/L), and/or thrombocytopenia (platelet count < 150 × 10^9^/L); pancreatitis; nausea; vomiting; anorexia; diarrhea; infections; flu-like symptoms; hypersensitivity reactions; skin manifestations; and headache. Other side effects reported as thiopurine induced were recorded as well.

### Statistical analysis

Efficacy was analyzed on both an intention-to-treat and per-protocol analysis basis. Continuous variables were reported as mean values and SDs or as medians and range depending on the normality of the underlying distribution. Continuous variables were consequently compared using the Wilcoxon signed-rank test or the Mann-Whitney *U* test, as appropriate. Categorical variables were presented as percentages and compared by using the χ^2^ test. A 2-sided *P* value of 0.05 or less was considered statistically significant. All data analyses were performed using IBM SPSS Statistics for Windows, version 24.0 (IBM).

### Ethics

The Research and Development Office of Great Ormond Street Hospital approved the review of clinical records for the research proposed in this study (Registration number: 2641).

## RESULTS

### Study population

Sixty-two children with IBD (male 32, median age at intervention 12 years, range 1–17 years) were included in the study. According to endoscopic and histological features, 36 (58%) patients were diagnosed with CD, 14 (23%) with IBD-U, and 12 (19%) with UC.

Fifty-seven patients (92%) had TPMT genotype tested before starting thiopurine treatment, with normal enzyme activity in 52 (91%). Fifty-seven of 62 patients (92%) received AZA, while the remaining patients were treated with 6-MP. The baseline demographics and disease-related characteristics of study patients are summarized in Table 1.

**Table 1.** Demographics and clinical characteristics at baseline

No. of cases	62
Gender male/female–number (%)	32/30
Age yr–median; IQR	14; 7
Diagnosis	
CD	36
UC	12
IBD-U	14
PCDAI–median (IQR)	5 (10)
PUCAI–median (IQR)	0 (6.25)
PGA whole cohort–numbers	
Remission	47/62
Mild	13/62
Moderate	2/62
Severe	0/62
PGA IBD-U—number	
Remission	9/14
Mild	3/14
Moderate	2/14
Severe	0/14
FC—median; IQR	330; 188
Medications—numbers	
Salicylates	23/62
Steroids	3/62
Biologics	18/62

CD, Crohn's disease; FC, fecal calprotectin; IBDU, IBDU, inflammatory bowel disease undefined; PCDAI, pediatric Crohn's disease activity index; PUCAI, pediatric ulcerative colitis activity index; UC, ulcerative colitis.

**Figure 1.**
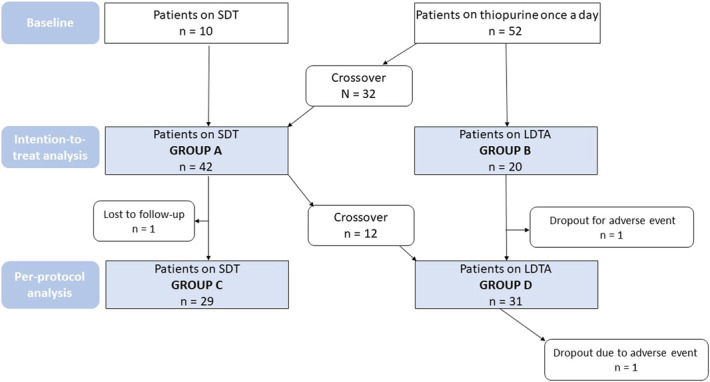
Flow chart of patients on split-dose regimen and thiopurine-allopurinol cotherapy and their crossover through the study.

At enrollment, 52 patients (84%) were started on thiopurine once a day, while 10 patients (16%) had been already started on SDT before referral to our department. During the study period, 32 of the 52 patients switched to SDT, resulting in a group of 42 patients (group A) and 20 patients switched to LDTA (group B). Based on therapeutic thiopurine monitoring performed at 6 months from the pharmacological intervention, 12 patients of 42 in the SDT group were crossed over to LDTA regimen due to persistent preferential 6-MeMP metabolism and 1 patient was lost to follow-up, resulting in a group of 29 patients on SDT treatment (group C). Of 20 patients receiving LDTA, 1 patient discontinued treatment because of an adverse reaction (headache) and 12 were switched from SDT, resulting in a group of 31 patients in LDTA (group D). Figure 1 shows the crossover of the patients throughout the study.

Reasons for switching treatment from once a day thiopurine monotherapy to STD or LDTA regimens were as follows: (i) raised 6-TGN/6-MeMP ratio (n = 37); (ii) high 6-TGN (n = 4); (iii) low 6-TGN (n = 7); (iv) abnormal liver function (n = 1); and (v) side effects related to thiopurine (n = 3).

### Efficacy

Using the intention-to-treat analysis, a comparison between group A and group B showed the treatment was effective in 29 of 42 children (69%) receiving SDT and 19 of 20 children (95%) receiving LDTA (*P* < 0.05). Indeed, on a per-protocol basis, no statistical difference was found between the treatment groups (group C [29/29; 100%] and group D [30/31; 97%]; *P* = NS).

At baseline, there was no statistically significant difference for 6-TGN/6-MeMP ratio between group A and group B (11.89 vs 13.21; *P* = 0.07), but 1–3 months postintervention, 6-TGN/6-MeMP ratio was significantly lower in group B compared with that in group A (0.67 vs 6.80; *P* < 0.001). Lower 6-TGN/6-MeMP ratio at 1–3 months compared with that at baseline was found for both groups (group A: 11.63 vs 13.95, *P* < 0.005; group B: 6.34 vs 0.64; *P* < 0.05) (see Supplementary Figure 1, http://links.lww.com/CTG/A894).

According to intention-to-treat analysis, there was a significant difference between group A and group B for TPMT (36 vs 41.5; *P* < 0.05), 6-TGN/6-MeMP ratio (6.80 vs 0.67; *P* < 0.001), 6-TGN (233 vs 334.5; *P* < 0.05), and 6-MeMP (1,298 vs 192; *P* < 0.001) at 1–3 months. There was no significant difference for white blood cells, neutrophils, lymphocytes, RBC, the mean corpuscular volume, platelet count, ALT, C-reactive protein, erythrocyte sedimentation rate, albumin, and fecal calprotectin (Table 2).

**Table 2.** Comparison between group A (split-dose regimen) and group B (thiopurine-allopurinol cotherapy) at baseline and 1–3 months postintervention

Variables (median; IQR)	Group A: SDT, n = 42	Group B: —LDTA, n = 20	*P* value
Baseline			
TPMT	36; 1.2	41.5; 4.2	<0.01
6-TGN	227.5; 20	198; 34.3	NS
6-MeMP	2,344; 335.3	2,397; 661.7	NS
6-TGN/6-MeMP RATIO	11.9; 61.1	13.2; 7.8	NS
ALT	21; 3.2	23; 7.3	NS
WBC	7.2; 0.4	7; 0.5	NS
Neutrophils	3.8; 0.4	3.5; 0.5	NS
Lymphocytes	1.9; 0.1	2.1; 0.2	NS
RBC	4.4; 0.1	4.3; 0.1	NS
MCV	82.4; 0.9	83.6; 4.1	NS
PLT	339; 17.2	320; 27.2	NS
CRP	5; 1.9	5; 1.2	NS
ESR	9.5; 3.4	21.5; 5.7	NS
Albumin	43; 0.7	45; 0.7	NS
Calprotectin	330; 190.4	253; 147.4	NS
1–3 mo			
6-TGN	233; 17.5	334.5; 36.5	<0.05
6-MeMP	1,298; 264.5	192; 320.5	<0.001
6-TGN/6-MeMP ratio	6.8; 0.9	0.7; 1.6	<0.001
ALT	20.5; 3.6	20.5; 2.3	NS
WBC	6.10; 0.3	6.4; 0.5	NS
Neutrophils	3.50; 0.3	3.1; 0.5	NS
Lymphocytes	1.70; 0.2	2.1; 0.2	NS
RBC	4.4; 0.1	4.2; 0.2	NS
PLT	315; 16.3	336; 25.4	NS
MCV	82.9; 0.9	84.8; 0.9	NS
CRP	5; 1.1	5; 1.1	NS
ESR	12; 3.6	23; 5.8	NS
Albumin	43; 0.7	45; 0.9	NS
Calprotectin	152.5; 254.7	346.5; 200.8	NS

6-MeMP, 6-methyl mercaptopurine; 6-TGN, 6-thioguanine nucleotide; alanine aminotransferase; ALT; CRP, C-reactive protein; ESR, erythrocyte sedimentation rate; LDTA, low dose azathioprine-allopurinol cotherapy; MCV, mean corpuscular volume; PLT, platelet count; RBC, red blood cell; SDT, split-dose thiopurine; TPMT, thiopurine methyltransferase; WBC, white blood cell.

As per protocol analysis, there was a significant difference between group D and group C at 6 months for 6-MeMP (262.5 vs 1,153.0; *P* < 0.05), 6-TGN/6-MeMP ratio (1.00 vs 5.00; *P* < 0.05), ALT (21.00 vs 15.50 U/L; *P* < 0.05), and platelet count (295.50 × 10^9^/L vs 352.50 × 10^9^/L; *P* < 0.05). At 12 months, only 6-MeMP (187.00 vs 854.50; *P* < 0.05) and 6-TGN/6-MeMP ratio (0.50 vs 3.50 *P* < 0.001) were significantly lower in group D compared with those in group C (Table 3).

**Table 3.** Comparison between group C (split-dose regimen) and group D (thiopurine-allopurinol cotherapy) at 6 and 12 months

Variables	Group C: SDT, n = 29	Group D: LDTA, n = 31	*P* value
6 mo			
6-TGN (median; IQR)	246.5; 22.7	298.5; 64.8	NS
6-MeMP (median; IQR)	1,153; 214.1	262.5; 192.9	<0.05
6-MeMP/6-TGN ratio (median; IQR)	5; 0.8	1; 1	<0.05
ALT (median; IQR)	15.50; 2.2	21; 6.5	<0.05
WBC (median; IQR)	6.2; 0.4	5.7; 0.5	NS
Neutrophils (median; IQR)	3.55; 0.3	2.85; 0.3	NS
Lymphocytes (median; IQR)	1.5; 0.2	2.3; 0.2	NS
RBC (median; IQR)	4.45; 0.1	4.35; 0.1	NS
MCV (median; IQR)	84; 1.5	85.3; 1.1	NS
PLT (median; IQR)	352.5; 14	295.5; 22.2	<0.05
CRP (median; IQR)	5; 1.4	4.9; 1	NS
ESR (median; IQR)	14.5; 6	7; 6.5	NS
Albumin (median; IQR)	43.5; 0.7	43; 0.8	NS
Calprotectin (median; IQR)	339.5; 162.2	104; 210.2	NS
Cotherapies (n)			
Infliximab	12	7	NS
Adalimumab	2	3	NS
5-ASA	5	12	NS
Steroids	0	0	—
12 mo			
6-TGN (median; IQR)	318; 32.7	322.5; 76.4	NS
6-MeMP (median; IQR)	854.5; 235.3	187.0; 126.2	<0.05
6-TGN/6-MeMP ratio (median; IQR)	3.50; 0.7	0.5; 0.2	<0.001
ALT (median; IQR)	15; 1.9	22; 1.5	NS
WBC (Median; IQR)	7.25; 0.3	6.90; 0.7	NS
Neutrophils (median; IQR)	4.05; 0.3	3.4; 0.5	NS
Lymphocytes (median; IQR)	2; 0.2	2.2; 0.2	NS
RBC (median; IQR)	4.55; 2.7	4.3; 0.4	NS
MCV (median; IQR)	82.95; 6.2	86.1; 1.3	NS
PLT (median; IQR)	365; 18.8	295; 13.4	NS
CRP (median; IQR)	5; 1.3	5; 0.9	NS
ESR (median; IQR)	11; 5.4	13; 2.4	NS
Albumin (median; IQR)	43; 0.8	43; 0.9	NS
Calprotectin (median; IQR)	378.5; 303.3	332.5; 289.4	NS
Cotherapies (n)			
Infliximab	11	7	NS
Adalimumab	2	4	NS
Vedolizumab	1	0	—
5-ASA	6	10	NS
Steroids	0	1	—

SDT, split-dose thiopurine; LDTA, low-dose azathioprine-allopurinol cotherapy; 6-TGN, 6-thioguanine nucleotide; 6-MeMP, 6-methyl mercaptopurine; ALT; alanine aminotransferase; WBC, white blood cell; RBC, red blood cell; MCV, mean corpuscular volume; PLT, platelet count; CRP, C-reactive protein; ESR, erythrocyte sedimentation rate.

Irrespective of the treatment group, significant lower levels of 6-TGN/6-MeMP ratio and 6-MeMP were found at 1–3 months, 6, and 12 months postintervention compared with those at baseline. Levels of 6-TGN were reduced at different time points, but without reaching statistically significant difference. ALT levels were significantly lower, whereas the mean corpuscular volume was significantly higher at 12 months compared with that at baseline. No significant difference was found between pediatric UC activity index/pediatric CD activity index and PGAI at baseline and after 12 months (Table 4).

**Table 4.** Comparison of continuous variables in whole cohort at different time points

Variables (median; IQR)	Baseline	1–3 mo	6 mo	12 mo
6-TGN	222; 19.4	297.5; 19.7	249.5; 25.9	327.5; 40.4
6-MeMP	2,433.5; 352.1	857.5; 191.1^a^	698; 188.4^a^	311; 190.4^a^
6-TGN/6-MeMP ratio	12.3; 1.1	2.8; 0.8^a^	3; 0.7^a^	1; 0.6^a^
CRP	5; 1.4	5; 0.8	5; 1	5; 0.9
ESR	13; 3	14; 3	10; 2	13; 3
Calprotectin	330; 188	136; 200	178; 104	182; 360
PGA whole cohort	1; 0	1; 0	1; 1	1; 1
PGA IBD-U	1; 1	1; 0	1; 1	1; 0
PCDAI	5; 10	2.5; 6.25	5; 10	0; 15
PUCAI	0; 6.25	0; 1.25	0; 15	0; 5

6-TGN, 6-thioguanine nucleotide; 6-MeMP, 6-methyl mercaptopurine; CRP, C-reactive protein; ESR, erythrocyte sedimentation rate; PGA, physician global assessment; IBDU, inflammatory bowel disease undefined; PCDAI, paediatric Crohn's disease activity index; PUCAI, paediatric ulcerative colitis activity index.

a *P* < 0.001.

### Adverse Events and Safety

Both treatment regimens were well tolerated because side effects reported were only few and not severe (Table 5). However, LDTA showed a relatively higher number of side effects compared with SDT regimen (20% vs 3%; *P* < 0.05). A discontinuation rate of 6% (2/32) was reported between patients tried on LTDA treatment (headache, n = 1; allergic reaction, n = 1).

**Table 5.** Side effects reported in the treatment groups during the study

Side effects	N
LDTA	
Allergic reaction	1
Low neutrophils	1
Abnormal pancreatic enzymes	1
Abnormal liver function	1
Vomiting	1
High 6-TGN	1
SDT	
Headache	1

6-TGN, 6-thioguanine nucleotide; LDTA, low-dose azathioprine-allopurinol cotherapy; SDT, split-dose thiopurine.

## DISCUSSION

This is the first study comparing the efficacy and the safety of SDT and LDTA regimens administered after failure of conventional thiopurine treatment in children with IBD. Our results showed that LDTA strategy is more effective than SDT in normalizing thiopurine metabolism in intention-to-treat analysis (95% vs 69%; *P* < 0.05), but was not superior to SDT regimen in per-protocol analysis (97% vs 100%; *P* = NS).

LDTA and SDT have been recently described as alternative strategies to overcome the preferential 6-MeMP metabolism reaching therapeutic 6-TGN levels (6,8,9). Recent guidelines on pediatric CD and UC from European Society of Pediatric Gastroenterology, Hepatology, and Nutrition (8,9) have also suggested LDTA as a promising strategy to harmonize thiopurine metabolite levels. However, only scant data are available in the literature about the use of LDTA in the pediatric population and whether its use is effective and safe in children has not been fully clarified yet.

In a retrospective study of a cohort of adults with IBD, Shih et al (6) first described that SDT reduces 6-MeMP levels and the associated 6-MeMP–related toxicities, while preserving the 6-TGN levels and without affecting clinical disease activity. The authors reported an efficacy rate for normalizing thiopurine of 60%. Our study showed similar results because 69% of patients (29 of 42 patients) responded to TSD strategy, while the remaining were switched to LDTA.

In a prospective study conducted on a small cohort of both adult and pediatric IBD, Gerich et al (13) observed that LDTA shifts metabolite production, reaching adequate therapeutic 6-TGN levels in 13 of 20 patients. Additional evidence on LDTA efficacy have been reported in a larger cohort of adults with IBD, in which the authors observed prospectively an efficacy rate of almost 90% (14). We found an efficacy of LDTA regimen ranging from 95% to 97%. The higher efficacy in pediatric population might be related to the correlation between age and xanthine oxidoreductase activity (15).

Although both strategies seemed to be safe, LDTA has showed higher occurrence of adverse reactions than SDT regimen in our study (20% vs 3%; *P* < 0.05). We also observed that LDTA induces a significant increase in ALT and a significant reduction in platelet count compared with SDT at 6 months. However, all the variables measured were within normal range and these changes did not persist at 12 months. The discontinuation rate due to adverse event of 6% (2 of 32) was reported in the LDTA arm of our study (allergic reaction, n = 1; headache, n = 1), while no patient dropped out the treatment in the SDT group. In a recent multicenter cohort study (16) conducted on patients older than 16 year who previously failed conventional thiopurines, the discontinuation rate due to adverse events of LDTA regimen in patients with IBD was 18%. The higher xanthine oxidoreductase enzymatic activity in adults might cause shunting toward the xanthine oxidoreductase pathway decreasing the bioavailability of 6MeMP toward transformation into 6TGN levels and increasing of toxic metabolites. That might explain the higher discontinuation rate in adults with IBD compared with pIBD (15). It should also be taken into account that in adult trials, allopurinol was used at 100 mg once daily, whereas in few pediatric case series, lower doses (50 or 75 mg once daily) were used in younger children (13,17,18). In our study, allopurinol dose of 50 mg daily in patients <30 kg or 100 mg daily in patients ≥30 kg was administered, accordingly to the European Society of Pediatric Gastroenterology, Hepatology and Nutrition guidelines (9). In our study, we could not evaluate whether the use of different allopurinol doses might be associated with adverse events.

It is worthy to emphasize that these treatments require careful and regular laboratory monitoring, especially in the first months of treatment; hence, it is advisable to manage them in centers with experienced IBD unit (9). The safety profile of thiopurines remains a topic of prominent consideration in pIBD, especially because a recent prospective study on a large cohort of pIBD concluded that thiopurines are an important risk factor for the development of malignancy and hemophagocytic lymphocytic histiocytosis (19). The impact of adding allopurinol to thiopurine therapy on malignancy or hemophagocytic lymphocytic histiocytosis risk has not been described and needs further studies.

Our study has some limitations inherent to retrospective assessment and lack of a standardized approach to clinical care. Furthermore, given the retrospective design of our study, some outcome measures to assess remission, such as PGA, have inherent limitations of subjectivity and are inferior to more objective measures used in clinical trials such as endoscopic or histologic assessment of mucosal inflammation.

Despite the aforementioned limitations, to the best of our knowledge, this is the first study comparing the efficacy and the safety of SDT and LDTA strategies in pIBD. Our study also gives an insight into the “real-world experience” of IBD clinical practice. For instance, due to the significant higher prevalence of side effects induced by LDTA, we are tempted to suggest that the administration of SDT should represent the first-line approach to reverse thiopurine shunting. LDTA could be a further option to use in patients failing SDT, but careful clinical and laboratory monitoring is warranted. It would be interesting in the future to evaluate predictor factors for SDT failure to tailor the most suitable treatment for each patient.

In conclusion, our study shows that both thiopurine split-dose administration and low-dose thiopurine-allopurinol cotherapy are effective therapeutic strategies in children with IBD and impaired thiopurine metabolism. Thiopurine-allopurinol cotherapy shows higher efficacy but also a higher rate of side effects, hence supporting the use of thiopurine split-dose as the first-line approach for reversing thiopurine shunting. Moreover, both strategies show sustained results over the time. Further prospective randomized studies are needed to confirm our observations.

## CONFLICTS OF INTEREST

**Guarantor of the article:** Osvaldo Borrelli, MD, PhD.

**Specific author contributions:** L.C.: preparation of synopsis data, analysis of results, drafting the manuscript, and approval of the final version of the paper. L.P.: analysis and interpretation of data, drafting the manuscript, critical revision of the manuscript, and approval of the final version of the paper. M.G.P.: preparation of synopsis data, drafting the manuscript, critical revision of the manuscript, and approval of the final version of the paper. S.E-K.: preparation of synopsis data, critical revision of the manuscript, and approval of the final version of the paper. S.C.: preparation of synopsis data, critical revision of the manuscript, and approval of the final version of the paper. R.B.: preparation of synopsis data, critical revision of the manuscript, and approval of the final version of the paper; E.G.: study design, recruitment of the patients; analysis and interpretation of data, critical revision of the manuscript, and approval of the final version of the paper. E.S.: analysis and interpretation of data, critical revision of the manuscript, and approval of the final version of the paper. F.K.: conception and study design, recruitment of patients, analysis and interpretation of data, critical revision of the manuscript, and approval of the final version of the paper. O.B.: conception and study design, analysis and interpretation of data, critical revision of the manuscript and approval of the final version of the paper.

**Financial support:** None to report.

**Potential competing interests:** None to report.

**Data availability statements:** Data underlying this article will be shared on reasonable request to the corresponding author.

**Study Highlights**

**WHAT IS KNOWN**

✓ Thiopurine is commonly prescribed for the maintenance of remission in children with inflammatory bowel disease.
✓ Its serum metabolites can be measured to monitor compliance and optimize levels.
✓ Where side effects occur or thiopurine metabolites shunt toward toxic levels, either low-dose azathioprine-allopurinol cotherapy or thiopurine split-dose has been suggested as pharmacological strategies to optimize serum concentrations.
✓ Which pharmacological intervention is more effective and safe in normalizing thiopurine serum levels in pediatric population with inflammatory bowel disease is not known.

**WHAT IS NEW HERE**

✓ Both thiopurine split-dose and low-dose thiopurine-allopurinol cotherapy are effective therapeutic strategies to optimize thiopurine levels in children with inflammatory bowel disease.
✓ Low-dose azathioprine-allopurinol cotherapy is more effective than split-dose thiopurine regimen in normalizing thiopurine metabolism but is associated with higher occurrence of adverse reactions.
